# First de novo whole genome sequencing and assembly of the bar-headed goose

**DOI:** 10.7717/peerj.8914

**Published:** 2020-04-06

**Authors:** Wen Wang, Fang Wang, Rongkai Hao, Aizhen Wang, Kirill Sharshov, Alexey Druzyaka, Zhuoma Lancuo, Yuetong Shi, Shuo Feng

**Affiliations:** 1 State Key Laboratory of Plateau Ecology and Agriculture, Qinghai University, Xi’ning, Qinghai, China; 2 Northwest Institute of Plateau Biology, Chinese Academy of Sciences, Xi’ning, Qinghai, China; 3 Novogene Bioinformatics Institute, Beijing, China; 4 College of Eco-Environmental Engineering, Qinghai University, Xi’ning, Qinghai, China; 5 Research Institute of Experimental and Clinical Medicine, Novosibirsk, Russia; 6 Institute of Systematics and Ecology of Animals, Siberian Branch of the Russian Academy of Sciences, Novosibirsk, Russia; 7 School of Finance and Economics, Qinghai University, Xi’ning, Qinghai, China; 8 KunLun College of Qinghai University, Xi’ning, Qinghai, China

**Keywords:** Bar-headed goose, Anser indicus, 10X Genomics Chromium, Avian genomes, Comparative genomics, Conservation genomics, High-altitude adaptation, Positive selection, Hypoxia, Qinghai-Tibetan Plateau

## Abstract

**Background:**

The bar-headed goose (*Anser indicus*) mainly inhabits the plateau wetlands of Asia. As a specialized high-altitude species, bar-headed geese can migrate between South and Central Asia and annually fly twice over the Himalayan mountains along the central Asian flyway. The physiological, biochemical and behavioral adaptations of bar-headed geese to high-altitude living and flying have raised much interest. However, to date, there is still no genome assembly information publicly available for bar-headed geese.

**Methods:**

In this study, we present the first de novo whole genome sequencing and assembly of the bar-headed goose, along with gene prediction and annotation.

**Results:**

10X Genomics sequencing produced a total of 124 Gb sequencing data, which can cover the estimated genome size of bar-headed goose for 103 times (average coverage). The genome assembly comprised 10,528 scaffolds, with a total length of 1.143 Gb and a scaffold N50 of 10.09 Mb. Annotation of the bar-headed goose genome assembly identified a total of 102 Mb (8.9%) of repetitive sequences, 16,428 protein-coding genes, and 282 tRNAs. In total, we determined that there were 63 expanded and 20 contracted gene families in the bar-headed goose compared with the other 15 vertebrates. We also performed a positive selection analysis between the bar-headed goose and the closely related low-altitude goose, swan goose (*Anser cygnoides*), to uncover its genetic adaptations to the Qinghai-Tibetan Plateau.

**Conclusion:**

We reported the currently most complete genome sequence of the bar-headed goose. Our assembly will provide a valuable resource to enhance further studies of the gene functions of bar-headed goose. The data will also be valuable for facilitating studies of the evolution, population genetics and high-altitude adaptations of the bar-headed geese at the genomic level.

## Introduction

High-altitude environments impose severe physiological challenges on vertebrates, owing to the decrease in oxygen, pressure, and temperature relative to lowland habitats. Understanding how vertebrates cope with these harsh conditions can provide important insights into the process of adaptive evolution (Zhang et al., 2016a; Zhang et al., 2016b). Among the high-altitude-adapted vertebrates, birds in general have excellent hypoxia tolerance and can maintain the highest basal metabolic rates during hypoxia too severe for most mammals to survive (Tucker, 1968; Faraci, 1991; Monge & Leon-Velarde, 1991), and are therefore especially compelling subjects for studies of high-altitude adaptation (Projecto-Garcia et al., 2013; Qu et al., 2013; Qu et al., 2015; Zhu et al., 2018).

One of the most well-known high-altitude bird species is the bar-headed goose (*Anser indicus*). Bar-headed geese breed in selected wetlands on the Qinghai-Tibetan Plateau (Takekawa et al., 2009) and Mongolian Plateau (Batbayar et al., 2014), and winter in the south-central Tibetan (Bishop et al., 1997) and India (Javed et al., 2000). The total worldwide populations of bar-headed geese were estimated at 97,000–118,000 individuals, with the highest numbers occurring in China (67.5–69.2%) and India (17.8–30.5%) (Liu et al., 2017). Bar-headed geese migrate along the central Asian flyway, and a certain proportion of individuals fly between central Asia (breeding areas) and India (wintering areas), and annually fly twice across the Himalayas (mean elevation 4,500 m) (Hawkes et al., 2011), where the partial pressure of oxygen is one-third of that at sea level (Scott & Milsom, 2007). Interestingly, bar-headed geese can sustain the high metabolic rates and the high rates of oxygen consumption needed for flapping flight in severe hypoxia during their migration across the Himalayas (Ward et al., 2002). Therefore, this species has become renowned for an example of high-altitude adaptation.

Many studies have sought to determine the physiological, molecular and behavioral basis for the successful adaptation of bar-headed geese to high-altitude flying and living (Butler, 2010; Scott et al., 2015). For example, bar-headed geese have evolved multiple mechanisms that enhance the uptake, circulation and peripheral diffusion of oxygen during hypoxia, including the increased lung mass and total ventilation (Scott & Milsom, 2007), hemoglobin with an increased oxygen affinity because of a single amino acid point mutation in the alpha polypeptide chain (McCracken, Barger & Sorenson, 2010; Natarajan et al., 2018), greater capillary density in flight and cardiac muscles increasing oxygen supply (Scott et al., 2009), and a higher proportion of mitochondria in a subsarcolemmal location reducing oxygen diffusion distances (Snyder, Byers & Kayar, 1984). In addition, bar-headed geese were found to take a roller coaster strategy, rising and falling with the underlying terrain, to conserve energy during Himalayan migrations (Bishop et al., 2015).

With the rapid evolution of genome sequencing technologies over the past decade, whole genome sequences could be obtained in a more economical and efficient way (Weisenfeld et al., 2017; Paajanen et al., 2019). For example, the high-quality reference genomes of the African wild dog (*Lycaon pictus*) were generated at a low cost by using the linked-read 10×  Genomics Chromium system (Armstrong Taylor et al., 2019). However, to date, there is still no genome assembly information publicly available for bar-headed geese. Ottenburghs et al. (2016) and Ottenburghs et al. (2017) sequenced nineteen goose genomes (including bar-headed goose) and used an exon-based phylogenomic approach (41,736 exons, representing 5,887 genes) to unravel the evolutionary history of geese group. However, the high quality of bar-headed goose genome assembly and annotation were unavailable in Ottenburghs et al. (2016) and Ottenburghs et al. (2017)’s work. In this study, we present the de novo whole genome sequencing and assembly of the bar-headed goose, along with gene prediction and annotation. We anticipate that these data will provide a valuable resource for future investigation of the genetic adaptation of bar-headed geese to high altitude, and particularly, for the comparative analysis of genome biology among several related birds (e.g., the pink-footed goose (*Anser brachyrhynchus*) (Pujolar et al., 2018), the swan goose (*Anser cygnoides*) (Lu et al., 2015) and the mallard (Huang et al., 2013)).

## Materials & Methods

### Ethics statement

This study conformed to the guidelines for the care and use of experimental animals established by the Ministry of Science and Technology of the People’s Republic of China (Approval number: 2006-398). The research protocol was reviewed and approved by the Ethical Committee of Qinghai University.

### Sampling and genome sequencing

A female bar-headed goose was collected in Huangzhong, Qinghai province of China, at an elevation of 3,000 m. Sampling (heart, liver, and lung) was done upon approved agreement of Xiulan Liu (20180411). The tissues were stored in liquid nitrogen and transported to the sequencing center (Novogene Bioinformatics Institute, Beijing, China). High-quality genomic DNA was extracted from the heart using the Qiagen DNA purification kit (Qiagen, Valencia, CA, USA) under the recommended protocol provided by the manufacturer. Standard 10X Genomic library was performed according to the manufacturer’s instructions described in the Chromium Genome User Guide Rev A (https://support.10xgenomics.com/de-novo-assembly/sample-prep/doc/user-guide-chromium-genome-reagent-kit-v1-chemistry). De novo sequencing was conducted with an Illumina HiSeq X Ten platform.

### Genome assembly and completeness evaluation

After removing adapter sequences and the unqualified sequences in the raw data, we de novo assembled the bar-headed goose genome from the high-quality clean reads using Supernova (version 1.1.3) (Weisenfeld et al., 2017). Supernova is a software package for de novo assembly from Chromium Linked-Reads that are made from a single whole-genome library from an individual DNA source (https://support.10xgenomics.com/de-novo-assembly/software/overview/latest/welcome). Standard assembly statistics were generated including: the length and number of contigs and scaffolds, number of scaffolds >2,000 bp, N50, N60, N70, N80 and N90, length of the longest contigs and scaffolds, and the total assembly length of contigs and scaffolds. The sequencing coverage and the guanine-cytosine (GC) content were also calculated.

After assembly, we evaluated the completeness of the bar-headed goose genome assembly using Core Eukaryotic Genes Mapping Approach software (CEGMA), which compared a set of 248 core eukaryotic genes to the assembled sequence (Parra, Bradnam & Korf, 2007). BUSCO analysis was also performed to assess the assembly quality by searching against the Benchmarking Universal Single-Copy Orthologs called BUSCOs (version 3.0) (Simao et al., 2015). In our study, the BUSCO dataset consisted of 2,586 single-copy orthologs genes.

### Genome annotation

First, protein-coding genes were predicted using three methods: homologous comparison, de novo prediction and RNA-seq based annotation. For the homology-based prediction, protein sequences from the other six avian species, including swan goose (*Anser cygnoides*) (Lu et al., 2015), ground tit (*Pseudopodoces humilis*) (Qu et al., 2013), red jungle fowl (*Gallus gallus*) (International Chicken Genome Sequencing Consortium, 2004), mallard (*Anas platyrhynchos*) (Huang et al., 2013), rock pigeon (*Columba livia*) (Shapiro et al., 2013) and zebra finch (*Taeniopygia guttata*) (Warren et al., 2010), were mapped onto the bar-headed goose genome, using TBLASTN with an *E*-value cutoff of 1e−5 (Altschul et al., 1997). Homologous genome sequences were aligned against the matching proteins using Genewise for accurate spliced alignments (Birney, Clamp & Durbin, 2004). We also used five tools of Augustus (Stanke & Morgenstern, 2005), GlimmerHMM (Majoros, Pertea & Salzberg, 2004), SNAP (Korf, 2004), GeneID (Guigo et al., 1992) and GeneScan (Burge & Karlin, 1997) for de novo prediction, in which the parameters were computationally optimized by training a set of high-quality proteins that have been derived from the Program to Assemble Spliced Alignment (PASA) gene models with default parameters (Campbell et al., 2006). RNA-seq data (manuscript in preparation) from three tissues (heart, liver, and lung) were also mapped to the bar-headed goose genome to further identify exon regions and splice positions using Tophat (-p 6 –max-intron-length 500,000 -m 2 –library-type fr-unstranded) (version 2.0.8) (Kim et al., 2013) and Cufflinks (-I 500,000 -p 1 –library-type fr-unstranded -L CUFF) (Trapnell et al., 2010). The final gene set was generated by merging all genes predicted by the three methods using EvidenceModeler (Haas et al., 2008).

Second, repetitive sequences in the bar-headed goose genome were identified using a combination of homology-based and de novo approaches. We used RepeatMasker and the associated RepeatProteinMask (Tarailo-Graovac & Chen, 2009) for homolog-based comparison by screening the published RepBase database (Bao, Kojima & Kohany, 2015). We used RepeatModeler (Smit & Hubley, 2018) to build a de novo repeat library. Then RepeatMasker was employed to align sequences from the bar-headed goose assembly to this *de novo* library for identifying repeat sequences. Tandem repeats, adjacent copies of a repeating pattern of nucleotides in the genomic sequences, were de novo predicted using Tandem Repeats Finder (TRF) tool (Benson, 1999).

Third, non-coding RNAs (tRNAs, rRNAs, miRNAs and snRNAs) were predicted. tRNA genes were identified using tRNAscan-SE (Schattner, Brooks & Lowe, 2005). rRNA genes were predicted by aligning to the rRNA sequences database using BlastN at *E*-value of 1E–10 (Altschul et al., 1990). MicroRNAs (miRNAs) and small nuclear RNAs (snRNAs) were identified by INFERNAL software (Nawrocki, Kolbe & Eddy, 2009) against the Rfam database (Kalvari et al., 2018) using the default settings.

Last, functional annotation of all genes was undertaken by BLASTP (evalue 1E–05). Protein domains were annotated by searching InterPro (version 32.0) (Mulder & Apweiler, 2007) and Pfam (Version 27.0) databases (Finn et al., 2014), using InterProScan (Version 4.8) and Hmmer (Potter et al., 2018) (Version 3.1) respectively. Gene Ontology terms for each gene were obtained from the corresponding InterPro or Pfam entry. The pathways, in which the gene might be involved, were assigned by blast against the KEGG database (release 53) (Ogata et al., 1999), with an *E*-value cutoff of 1E–05.

### Comparative genome analysis

Gene families were identified using TreeFam (Li et al., 2006) with default settings from 16 animal genomes [bar-headed goose (*Anser indicus*), swan goose (*Anser cygnoides*) (Lu et al., 2015), crested ibis (*Nipponia nippon*) (Li et al., 2014), mallard (*Anas platyrhynchos*) (Huang et al., 2013), red junglefowl (*Gallus gallus*) (International Chicken Genome Sequencing Consortium, 2004), turkey (*Meleagris gallopavo*) (Dalloul et al., 2010), common cuckoo (*Cuculus canorus*) (https://www.ncbi.nlm.nih.gov/assembly/GCF_000709325.1/), rock pigeon (*Columba livia*) (Shapiro et al., 2013), ground tit (*Pseudopodoces humilis*) (Qu et al., 2013), bananaquit (*Coereba flaveola*) (Antonides, Ricklefs & DeWoody, 2017), great tit (*Parus major*) (Qu et al., 2015), zebra finch (*Taeniopygia guttata*) (Warren et al., 2010), ruff (*Philomachus pugnax*) (Lamichhaney et al., 2016), peregrine falcon (*Falco peregrinus*) (Zhan et al., 2013), yak (*Bos mutus*) (Qiu et al., 2012) and tibetan antelope (*Pantholops hodgsonii*) (Ge et al., 2013)]. A total of 2,888 single-copy orthologs, shared by these 16 species, were finally identified. These single-copy orthologs were then subjected to BLAST searches against all genomes using MUSCLE software, applying the default search parameters (Edgar, 2004). CAFE (De Bie et al., 2006) was employed to detect gene families that have undergone expansion or contraction in the bar-headed goose compared with the other 15 species. The GO enrichment analysis was then applied to identify the significantly enriched biological process of the expanded or contracted gene families (*P* < 0.01, FDR (false discovery rate) <0.05). The above single-copy orthologs were also employed to detect genes evolving under positive selection in the bar-headed goose compared with swan goose. We used the codeml program within PAML to calculate the ratio of the non-synonymous to synonymous substitutions per gene (Yang, 2007). A likelihood ratio test was performed to identify the positively selected genes (PSGs) using FDR correction to adjust the *P*-value (cutoff: 0.05).

### Data access

The sequencing data in the fastq format have been deposited in NCBI Sequence Read Archive (SRA) with accession number SRP199943 and Bioproject accession PRJNA542959. The assembled draft genome has been deposited at DDBJ/ENA/GenBank under the accession VDDG00000000. The version described in this paper is version VDDG01000000. The annotation results of repeated sequences, gene structure, non-coding RNAs and functional prediction were deposited in Figshare database (https://doi.org/10.6084/m9.figshare.8229083.v1).

## Results

### Genome sequencing, assembly and annotation

We sequenced the genome of a female bar-headed goose from Huangzhong, Qinghai, China using the linked-read 10×  Genomics Chromium system and the Illumina HiSeq X Ten platform. We obtained a total of 124 Gb sequencing data (∼103-fold coverage) with a read length of 150 bp and an insert size of 600 bp (Table S1). We then used these data to de novo assemble the bar-headed goose genome using the 10×  Genomics Supernova assembler. The final total assembled genome length was 1.14 Gb with a contig N50 length of 120 Kb, and a scaffold N50 length of 10.09 Mb (Table 1). The average GC content of the bar-headed goose genome was 41.87% (Table S2). CEGMA and BUSCO analyses were performed to assess the completeness of the assembled bar-headed goose genome. Using CEGMA on our assembly, we found that 211 conserved genes (85.08%) in bar-headed goose genome were successfully assembled, when compared to the 248 evolutionarily conserved core gene sets from six eukaryotic model organisms (Table S3). According to BUSCO analysis, 97.5% of the 2,586 conserved genes were identified in our assembled bar-headed goose genome (Table 2). These results indicated that the assembly of the bar-headed goose genome showed a high level of completeness. We detected 8.9% repeat sequences in bar-headed goose genome (Table S4), including long interspersed nuclear elements (LINEs, 6.20%), long terminal repeats (LTRs, 1.73%), and DNA transposons 0.27%. We also predicted 342 microRNAs (miRNAs), 49 rRNAs, 282 tRNAs, and 288 small nuclear RNAs (snRNAs) in the bar-headed goose genome (Table 3). By combining homologous comparison, de novo prediction and RNA-seq assisted methods, we predicted 16,428 protein-coding genes in the bar-headed goose genome (Table 4). The average length of genes were 25,274 bp with an average of 10 exons per gene (Table 4). Of these protein-coding genes, a total of 15,790 genes (96.1%) were functionally annotated according to NCBI’s Reference Sequence (RefSeq) database (O’Leary et al., 2016), Swiss-prot, KEGG and InterPro databases (Table 5).

**Table 1:** The de novo assembled genome of Bar-headed goose.

	Length (bp)		Numbers	
	Contigs	Scaffolds	Contigs	Scaffolds
Total	1,114,495,510	1,143,097,520	30,886	10,528
Max	1,384,698	33,819,004	–	–
Number ≥ 2000	–	–	22,660	5,540
N50	120,377	10,094,206	2,510	35
N60	91,372	8,026,496	3,576	48
N70	65,644	5,883,601	5,019	64
N80	44,047	3,435,270	7,086	90
N90	23,142	1,267,474	10,506	144

**Table 2:** The BUSCO assessment results of the completeness of genome assembly.

Species	BUSCO assessment results
Bar-headed goose	C: 97.5%, [D: 0.4%], F: 1.7%, M: 0.8%, n: 2586

**Notes.**

C Complete Single-Copy BUSCOs D Complete Duplicated BUSCOs F Fragmented BUSCOs M Missing BUSCOs n Total BUSCO groups searched

**Table 3:** Annotation of non-coding RNA genes.

Type		Copy	Average length (bp)	Total length (bp)	% of genome
miRNA		342	85.98	29,406	0.003
tRNA		282	75.09	21,175	0.002
rRNA	**rRNA**	49	234.67	11,499	0.001
	18S	9	426.89	3,842	0.000
	28S	33	211.39	6,976	0.001
	5.8S	2	156.00	312	0.000
	5S	5	73.80	369	0.000
snRNA	**snRNA**	288	118.40	34,099	0.003
	CD-box	106	86.92	9,214	0.001
	HACA-box	80	139.41	11,153	0.001
	splicing	84	128.12	10,762	0.001

**Table 4:** Prediction of protein-coding genes.

				Average length (bp)			
Methods		Gene number	Exons per gene	Gene	CDS	Exon	Intron
De novo	Augustus	18,318	8.14	16,402.87	1,402.91	172.29	2,099.99
	GlimmerHMM	172,168	2.90	5,695.00	474.06	163.45	2,747.47
	SNAP	61,271	6.31	29,996.61	809.23	128.21	5,495.13
	Geneid	38,214	5.48	20,392.89	1,006.18	183.58	4,326.65
	Genscan	43,335	7.61	19,762.90	1,294.69	170.05	2,792.52
Homologous comparison	*Taeniopygia guttata*	26,141	5.64	13,827.51	1,049.69	186.24	2,756.00
	*Anas platyrhynchos*	36,335	4.65	9,142.51	930.19	199.91	2,248.05
	*Gallus gallus*	25,943	6.03	13,297.66	1,171.56	194.16	2,408.92
	*Columba livia*	36,315	4.71	9,177.85	913.73	194.05	2,228.25
	*Anser cygnoides*	34,171	5.14	10,493.05	1,004.18	195.43	2,292.94
	*Pseudopodoces humilis*	15,498	9.06	21,225.26	1,648.10	181.90	2,428.77
RNA-seq	Cufflinks	48,064	7.93	24,121.46	3,494.75	440.7	2,976.44
	PASA	65,640	6.78	15,650.31	1,168.16	172.35	2,506.53
EVM		24,169	7.12	16,340.12	1,225.82	172.14	2,469.26
PASA-update		24,010	7.20	17,772.08	1,249.46	173.43	2,663.08
Final set		16,428	9.88	25,274.02	1,641.91	166.26	2,662.69

**Table 5:** Functional annotation of the predicted protein-coding genes.

Database		Number	Percent (%)
RefSeq		15,780	96.1
Swiss-Prot		15,287	93.1
KEGG		13,802	84.0
InterPro	All	15,106	92.0
	Pfam	13,875	84.5
	GO	11,272	68.6
Annotated		15,790	96.1
Total		16,428	–

### Comparative genomic analysis

**Figure 1:**
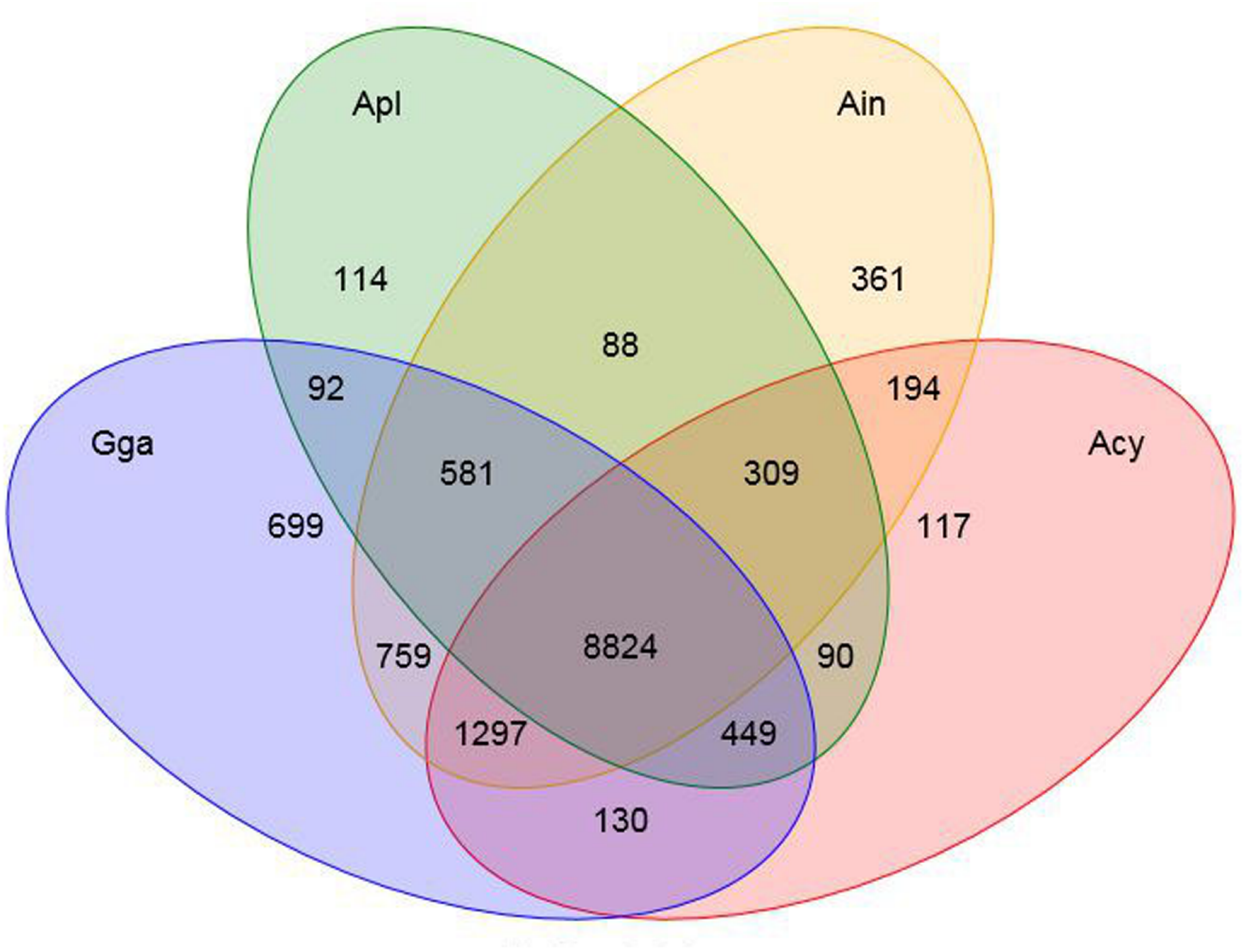
Orthologous genes in bar-headed goose and other birds. The number of unique or shared orthologous genes are listed in each diagram component. Ain, bar-headed goose; Acy, swan goose; Apl, mallard; Gga, red junglefowl.

We identified a total of 18,914 common gene families from the 16 available vertebrates’ genomes, including swan goose (*Anser cygnoides*, Acy), crested ibis (*Nipponia nippon*, Nni), mallard (*Anas platyrhynchos*, Apl), red junglefowl (*Gallus gallus*, Gga), turkey (*Meleagris gallopavo*, Mga), common cuckoo (*Cuculus canorus*, Cca), rock pigeon (*Columba livia*, Cli), ground tit (*Pseudopodoces humilis*, Phu), bananaquit (*Coereba flaveola*, Cfl), great tit (*Parus major*, Pma), zebra finch (*Taeniopygia guttata*, Tgu), ruff (*Philomachus pugnax*, Ppu), peregrine falcon (*Falco peregrinus*, Fpe), yak (*Bos mutus*, Bmu) and tibetan antelope (*Pantholops hodgsonii*, Pho), of which 2,888 represented single-copy orthologous genes (Fig. S1). Furthermore, the number of unique or shared orthologous genes among bar-headed goose, swan goose, mallard and red junglefowl was provided by a Venn diagram (Fig. 1). Of these four species, 361 orthologous genes were found to be unique in the bar-headed goose genome. 50% and 27.32% of these specific orthologous genes were enriched in gene ontology (GO) terms related to molecular functions and biological process, respectively. The molecular functions were involved in protein binding (GO: 0005515) and ATP binding (GO: 0005524), while the biological process was involved in signal transduction (GO: 0007165) and G-protein coupled receptor signaling pathway (GO: 0007186) (Table S5).

### Expansion and contraction of gene families

**Figure 2:**
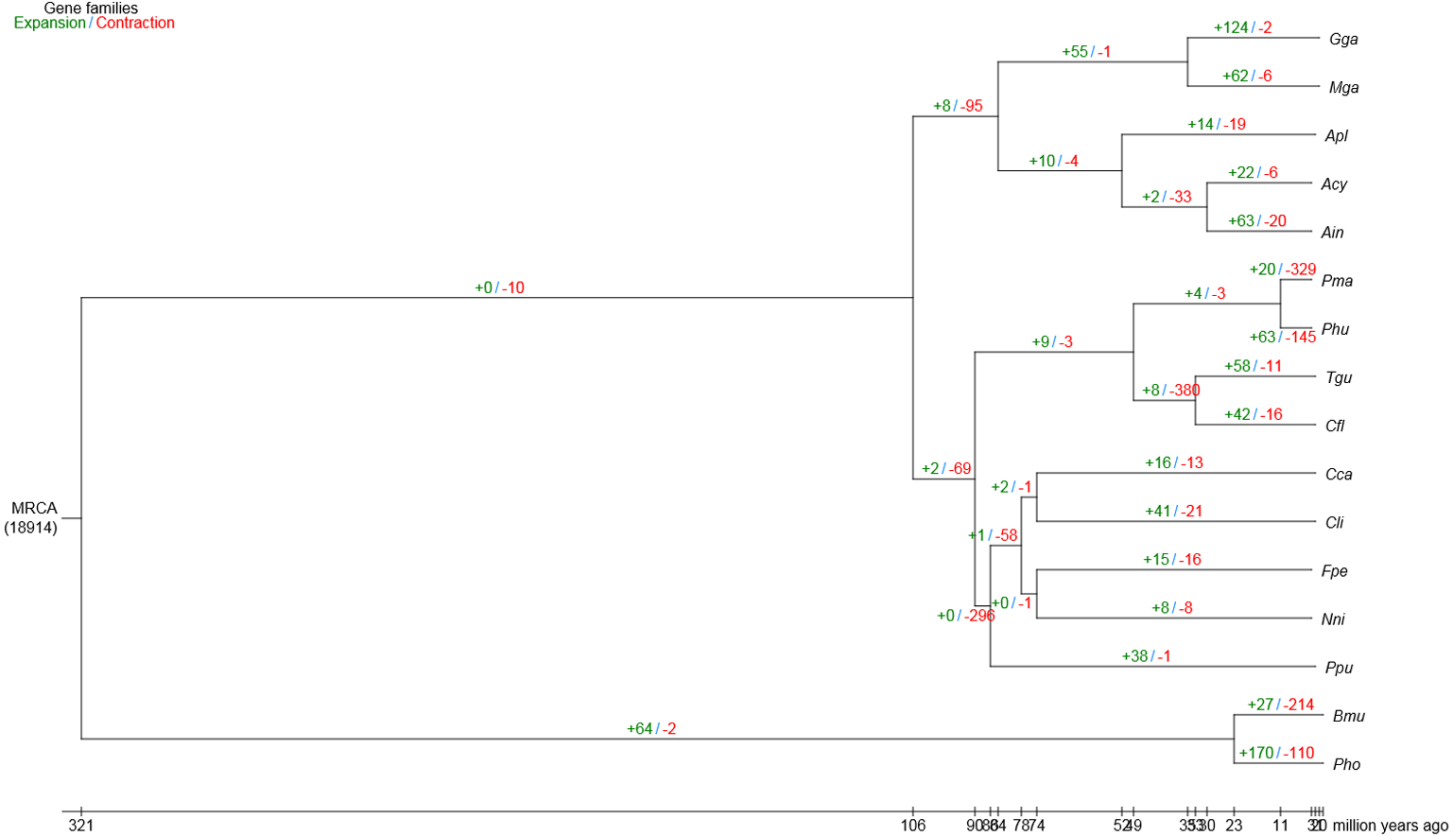
Gene family expansion and contraction in the bar-headed goose genome. The number of expanded (blue) and contracted (red) gene families are shown along branches and nodes. MRCA, most recent common ancestor; Ain, bar-headed goose; Acy, swan goose; Nni, crested ibis; Apl, mallard; Gga, red junglefowl; Mga, turkey; Cca, common cuckoo; Cli, rock pigeon; Phu, ground tit; Cfl, bananaquit; Pma, great tit; Tgu, zebra finch; Ppu, ruff; Fpe, peregrine falcon; Bmu, yak; Pho, tibetan antelope.

Compared with the other 15 vertebrates, we identified 63 and 20 gene families that were substantially expanded and contracted in the bar-headed goose, respectively (Fig. 2). Functional identities were presented for all significantly expanded and contracted gene families in Tables S6 and S7, respectively. Most of the significantly expanded gene families were associated with the functional categories of adhesion, transport, hydrolase activities and binding (Table S6). Examples of expanded gene families in the adhesion functional class included homophilic cell adhesion (GO: 0007156), cell–cell adhesion (GO: 0098609) and biological adhesion (GO: 0022610), among others. The transport functional class of expanded gene families included urea transport (GO: 0015840), one-carbon compound transport (GO: 0019755), monovalent inorganic cation transport (GO: 0015672), amide transport (GO: 0042886) and transmembrane transporter complex (GO: 1902495), among others. Specific examples of expanded gene families that belong to the hydrolase activities functional class included metalloendopeptidase activity (GO: 0004222), endopeptidase activity (GO: 0004175), phosphorylase activity (GO: 0004645) and helicase activity (GO: 0004386), among others. The binding functional class of expanded gene families included ion binding (GO: 0043167), ATP binding (GO: 0005524), protein binding (GO: 0005515), adenyl nucleotide binding (GO: 0030554) and Rho GTPase binding (GO: 0017048), among others. For gene families that were significantly contracted, the main functional categories, as indicated by GO annotations, also included adhesion, transport, hydrolase activities and binding (Table S7). However, gene family members belonging to the specific GO terms that showed contraction differed from those that showed significant expansion.

### Positive selection in bar-headed goose

**Figure 3:**
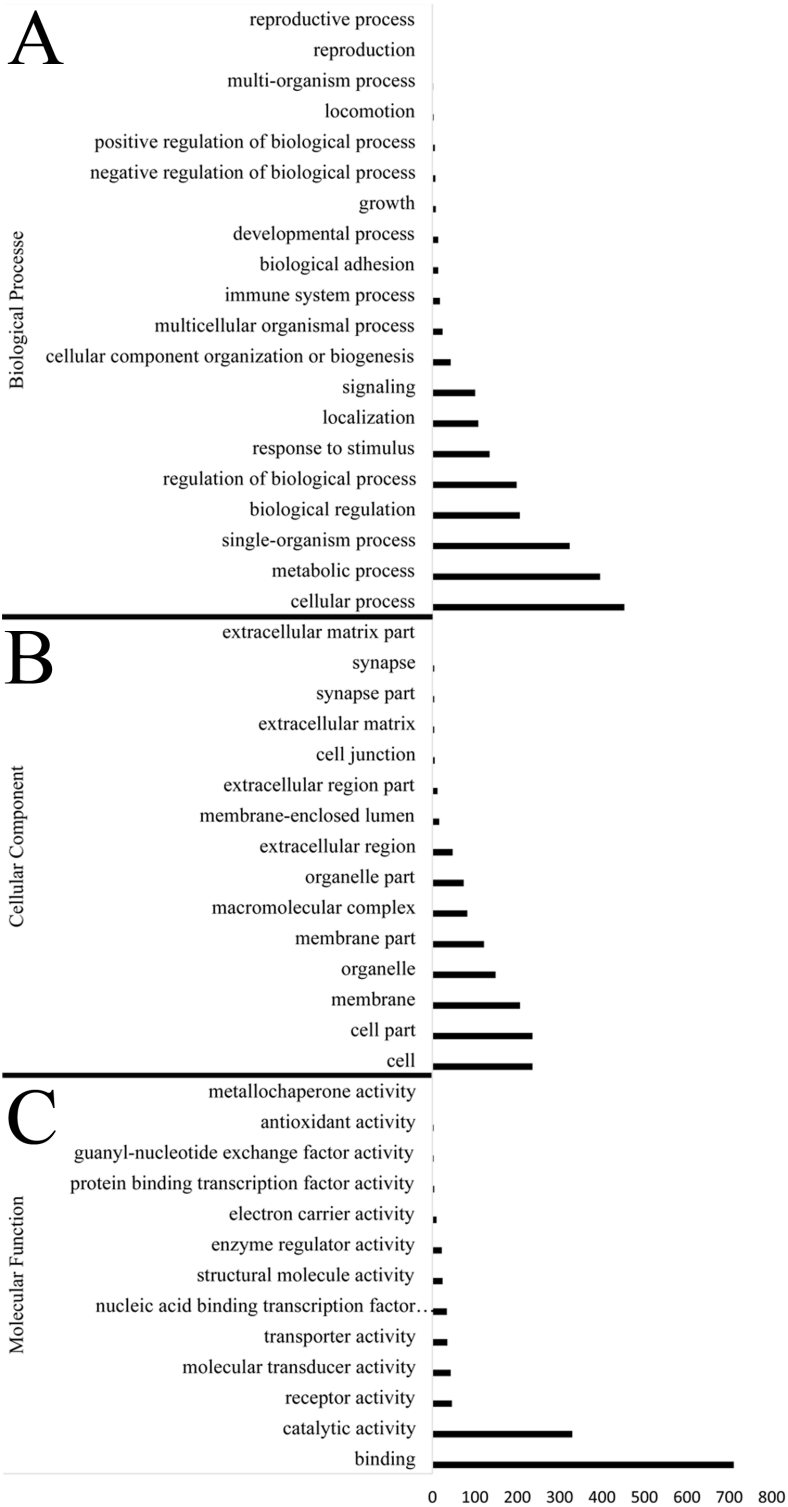
Functional distribution of positively selected genes (PSGs) according to the Gene Ontology (GO) database. The *y* axis reveals the GO functional categories, including (A) biological process, (B) cellular component, and (C) molecular function, while the number of genes in each category is plotted on the *x* axis.

Identification of genes subject to positive selection in the bar-headed goose genome is key to understand its adaptation to high-altitude environments. Compared with swan goose, a relative species living at low altitude areas, we identified 1,715 positively selected genes (PSGs) in the bar-headed goose genome using the branch-site likelihood ratio test (Table S8). The GO annotation for these PSGs was shown in Fig. 3. Molecular functions contained genes mainly involved in binding (711 genes, GO: 0005488) and catalytic activity (330 genes, GO: 0003824). Genes associated with cellular components were primarily cell (236 genes, GO: 0005623), cell part (236 genes, GO: 0044464), membrane (207 genes, GO: 0016020) and organelle (149 gene, GO: 0043226). Genes related to biological process were mainly involved in cellular process (453 genes, GO: 0009987), metabolic process (396 genes, GO: 0008152), single-organism process (324 genes, GO: 0044699), biological regulation (206 genes, GO: 0065007) and response to stimulus (135 genes, GO: 0050896). These different functional categories resulting from the GO annotations indicated that a substantial diversity of PSGs were present in the bar-headed goose genome.

We found several PSGs related to the adaptation of bar-headed goose to the harsh high-altitude environment. For example, there were nine PSGs (*IFNGR2*, *CDKN1B*, *PDHA1*, *EIF4E2*, *EP300*, *IFNG*, *NPPA*, *PIK3R5* and *MAPK1*) annotated in the HIF-1 (Hypoxia-Inducible Factor-1) signaling pathway (map04066). Several PSGs associated with response to ultraviolet (UV) radiation were annotated in the cellular response to DNA damage stimulus (16 genes, GO: 0006974) and the DNA repair (15 genes, GO: 0006281). PSGs, which were annotated in the ATP binding (79 genes, GO: 0005524), GTP binding (29 genes, GO: 0005525) and endoplasmic reticulum (10 genes, GO: 0005783), were related to the energy metabolism rate under hypoxic conditions. In addition, we also found several other PSGs, which might be involved in response to high-altitude environment. *VASH2*, annotated in angiogenesis (GO: 0001525), might be a mediator in hypoxia-induced angiogenesis. *PGR* gene annotated in steroid hormone receptor activity (GO: 0003707), which might play a key role in diverse events associated with reproduction in female geese under the high-altitude condition. The positively selected *MYOG* (annotated in GO: 0007517) and *TNNT3* (annotated in GO: 0006936) might have important implications in the skeletal development. However, all these genes need to be validated by future functional experiments.

## Discussion

The number of bird genomes accessible has increased dramatically during the last couple of years due to the Bird 10,000 Genomes (B10K) Project (https://b10k.genomics.cn), which was aimed to generate representative draft genome sequences from all extant bird species (Zhang et al., 2015). Comparative genomic analyses of 48 consistently annotated bird genomes from the first phase of the B10K project provided new perspectives on avian evolution, ecology and conservation (Jarvis et al., 2014; Zhang et al., 2014). With the decrease in sequencing costs and improvements in genome assembly algorithms, many more bird genome sequences will be published at a striking pace (Ekblom & Wolf, 2014; Kraus & Wink, 2015). Bar-headed goose, a species occupying a wide variety of habitats on the Qinghai-Tibetan Plateau, is considered a good model for studying high-altitude adaptation in birds. Much of what is known about the mechanisms of high-altitude adaptation in bar-headed geese comes from studies that have taken physiological, biochemical, and morphological methods (Snyder, Byers & Kayar, 1984; Scott & Milsom, 2007; Scott et al., 2009; *Butler, 2010; McCracken, Barger & Sorenson, 2010*; *Scott et al., 2015; Natarajan et al., 2018)*. An understanding of the genetic basis of this adaptation, however, has lagged behind due to the unavailability of the bar-headed goose genome. Therefore, we sequenced, assembled, and annotated a bar-headed goose genome for the first time to provide a valuable tool for future population genetics and comparative genomics studies associated with this species.

Birds have the smallest genomes among amniotes, ranging from an estimated 0.9 Gb to 2.1 Gb (Gregory, 2019). In the current study, the genome size of the bar-headed goose was estimated to be 1.2 Gb and a total of 124 Gb (∼103-fold coverage) sequencing data was generated. The assembled sequence was 1.143 Gb, consisting of 10,528 scaffolds with a scaffold N50 of 10.09 Mb. In comparison with the swan goose genome (Lu et al., 2015) and pink-footed goose genome (Pujolar et al., 2018), our bar-headed goose genome exhibits longer contig N50 lengths and scaffold N50 lengths. The assembly was well supported by the BUSCO and CEGMA assessments. The average GC content of the bar-headed goose genome was around 41.87%, similar to that of the other birds such as Sichuan white goose (*Anser cygnoides Linn. var domestica*) (Gao et al., 2016) and buff-throated partridge (*Tetraophasis szechenyii*) (Zhou et al., 2019). Repeat sequences comprised approximately 8.9% of the bar-headed goose genome, which was higher than that of the Sichuan white goose genome at 6.9%. These results were consistent with previous reports indicating that almost all avian genomes present lower levels of repeat elements (∼4 to 10% of each genome) than those of other tetrapod vertebrates (for example, 34 to 52% in mammals) (Bohne et al., 2008; Kapusta & Suh, 2017). We predicted 16,428 protein-coding genes in the bar-headed goose, 96.1% of which were functionally annotated by public databases. The gene number of bar-headed goose was intermediate between the gene numbers of swan goose (16,150 genes) and pink-footed goose (26,134 genes).

Furthermore, a gene family clustering of four bird species (bar-headed goose, swan goose, mallard and red junglefowl) from the three clades within the phylogenetic tree identified 361 unique orthologous genes in the bar-headed goose genome comparing with the other three species. We then examined large-scale differences in gene families within 14 species of birds and between birds and two mammals using the genome data. In total, we determined that there were 63 expanded and 20 contracted gene families in the bar-headed goose compared with the other 15 vertebrates. The detailed evolutionary history and the function of each gene family needs further investigation. We performed a positive selection analysis between the bar-headed goose and the closely related low-altitude goose, swan goose, to uncover its genetic adaptations to the Qinghai-Tibetan Plateau. The main stressor at high altitude is hypoxia. Most responses to hypoxia are mediated by the activation of a family of transcription factors named hypoxia-inducible factors (HIFs). In hypoxic conditions, HIFs are stabilized and enter the nucleus, where they bind to the Hypoxia Response Elements (HRE) and finally induce transcription of a large number of target genes. In our study, nine PSGs were annotated in the HIF-1 (Hypoxia-Inducible Factor-1) signaling pathway. These genes may be important for the bar-headed goose to survive in the high-altitude environment. For example, elevated expression of *CDKN1B* (p27Kip1) during hypoxia could arrest cells in G_0_/G_1_ phase and may prevent the inappropriate proliferation of genetically damaged cells (Kumar & Vaidya, 2016). Pyruvate dehydrogenase (PDH) occupies a central crossroad between glycolysis and the tricarboxylic acid cycle. It was reported that cardiac *PDHA1* (Pyruvate Dehydrogenase E1 Alpha 1 Subunit) deficiency impairs ischemic AMP-activated protein kinase signaling and sensitizes hearts to the toxicological actions of ischemic stress (Sun et al., 2016). *EP300* (E1A Binding Protein P300), functions as a histone acetyltransferase, was identified as a co-activator of HIF-1A, and thus plays a role in the stimulation of hypoxia-induced genes (Dames et al., 2002). Natriuretic peptides are a well-described family of hormones with a major role in sodium and body volume homeostasis through their actions on renal hemodynamics and tubular function (Nakao et al., 1996). These positively selected genes in the bar-headed goose genome laid a solid foundation for understanding the specific adaptations to the high-altitude environments, in addition to other characteristics known from other high-altitude bird species (Scott, 2011; Qu et al., 2013). The functional role of these genes can be investigated in the future.

## Conclusions

In summary, this is the first report describing the sequencing, assembly, and annotation of the bar-headed goose genome and contributes to the genomic resources for studying its genome evolution and the adaptive mechanisms that confer its resistance to the harsh environments on the Qinghai-Tibetan Plateau. Functional experimental assays will be needed to validate the actual functional significance of many of the positively selected genes.

##  Supplemental Information

10.7717/peerj.8914/supp-1 Figure S1 The distribution of orthologous in the bar-headed goose and other vertebrates Ain: bar-headed goose. Acy: swan goose. Nni: crested ibis. Apl: mallard. Gga: red junglefowl. Mga: turkey. Cca: common cuckoo. Cli: rock pigeon. Phu: ground tit. Cfl: bananaquit. Pma: great tit. Tgu: zebra finch. Ppu: ruff. Fpe: peregrine falcon. Bmu: yak. Pho: tibetan antelope.

10.7717/peerj.8914/supp-2 Table S1 Summary statistics for the sequencing data

10.7717/peerj.8914/supp-3 Table S2 GC content of the Bar-headed goose genome

10.7717/peerj.8914/supp-4 Table S3 The CEGMA assessment results of the completeness of genome assembly Complete: more than 70% of the core genes were assembled. Complete + partial: partial of the core genes were assembled. #Prots: the number of the assembled core genes. %Completeness: the ratio of the assembled core genes / the whole core gene sets.

10.7717/peerj.8914/supp-5 Table S4 Annotation of repeated sequences

10.7717/peerj.8914/supp-6 Table S5 GO enrichment in specific orthologous gene families found exclusively in the Bar-headed goose

10.7717/peerj.8914/supp-7 Table S6 Functional annotation for gene families showing significant expansion in the Bar-headed goose genome

10.7717/peerj.8914/supp-8 Table S7 Functional annotation for gene families showing significant contraction in the Bar-headed goose genome

10.7717/peerj.8914/supp-9 Table S8 Positively selected genes identified in the Bar-headed goose genome

## Additional Information and Declarations

### Field Study Permissions

The following information was supplied relating to field study approvals (i.e., approving body and any reference numbers): Xiulan Liu granted access to her private field for the the sample collection (20180411).

### Data Availability

The following information was supplied regarding data availability: The sequencing data are available in NCBI Sequence Read Archive (SRA): (SRP199943) and Bioproject (PRJNA542959). The assembled draft genome is available at DDBJ/ENA/GenBank: VDDG00000000. The version in this article is VDDG00000000. The annotation results of repeated sequences, gene structure, non-coding RNAs and functional prediction are available in FigShare: Wang, Wen (2019): Annotation results of Bar-headed goose genome. figshare. Online resource. https://doi.org/10.6084/m9.figshare.8229083.v1.

## References

[ref-1] Altschul SF, Gish W, Miller W, Myers EW, Lipman DJ (1990). Basic local alignment search tool. Journal of Molecular Biology.

[ref-2] Altschul SF, Madden TL, Schaffer AA, Zhang J, Zhang Z, Miller W, Lipman DJ (1997). Gapped BLAST and PSI-BLAST: a new generation of protein database search programs. Nucleic Acids Research.

[ref-3] Antonides J, Ricklefs R, DeWoody JA (2017). The genome sequence and insights into the immunogenetics of the bananaquit (Passeriformes: Coereba flaveola). Immunogenetics.

[ref-4] Armstrong EE, Taylor RW, Prost S, Blinston P, Van der Meer E, Madzikanda H, Mufute O, Mandisodza-Chikerema R, Stuelpnagel J, Sillero-Zubiri C, Petrov D, Dmitri P (2019). Cost-effective assembly of the African wild dog (Lycaon pictus) genome using linked reads. Gigascience.

[ref-5] Bao W, Kojima KK, Kohany O (2015). Repbase update, a database of repetitive elements in eukaryotic genomes. Mobile DNA.

[ref-6] Batbayar N, Takekawa JY, Natsagdorj T, Spragens KA, Xiao X (2014). Site selection and nest survival of the bar-headed goose (Anser indicus) on the Mongolian plateau. Waterbirds.

[ref-7] Benson G (1999). Tandem repeats finder: a program to analyze DNA sequences. Nucleic Acids Research.

[ref-8] Birney E, Clamp M, Durbin R (2004). GeneWise and genomewise. Genome Research.

[ref-9] Bishop CM, Spivey RJ, Hawkes LA, Batbayar N, Chua B, Frappell PB, Milsom WK, Natsagdorj T, Newman SH, Scott GR, Takekawa JY, Wikelski M, Butler PJ (2015). The roller coaster flight strategy of bar-headed geese conserves energy during Himalayan migrations. Science.

[ref-10] Bishop MA, Song YL, Canjue ZM, Gu BY (1997). Bar-headed geese Anser indicus wintering in South-central Tibet. Wildfowl.

[ref-11] Bohne A, Brunet F, Galiana-Arnoux D, Schultheis C, Volff JN (2008). Transposable elements as drivers of genomic and biological diversity in vertebrates. Chromosome Research.

[ref-12] Burge C, Karlin S (1997). Prediction of complete gene structures in human genomic. Journal of Molecular Biology.

[ref-13] Butler PJ (2010). High fliers: the physiology of bar-headed geese. Comparative Biochemistry and Physiology. Part A, Molecular and Integrative Physiology.

[ref-14] Campbell MA, Haas BJ, Hamilton JP, Mount SM, Buell CR (2006). Comprehensive analysis of alternative splicing in rice and comparative analyses with Arabidopsis. BMC Genomics.

[ref-15] Dalloul RA, Long JA, Zimin AV, Aslam L, Beal K, Blomberg LA, Bouffard P, Burt DW, Crasta O, Crooijmans RP, Cooper K, Coulombe RA, De S, Delany ME, Dodgson JB, Dong JJ, Evans C, Frederickson KM, Flicek P, Florea L, Folkerts O, Groenen MA, Harkins TT, Herrero J, Hoffmann S, Megens HJ, Jiang A, De Jong P, Kaiser P, Kim H, Kim KW, Kim S, Langenberger D, Lee MK, Lee T, Mane S, Marcais G, Marz M, McElroy AP, Modise T, Nefedov M, Notredame C, Paton IR, Payne WS, Pertea G, Prickett D, Puiu D, Qioa D, Raineri E, Ruffier M, Salzberg SL, Schatz MC, Scheuring C, Schmidt CJ, Schroeder S, Searle SM, Smith EJ, Smith J, Sonstegard TS, Stadler PF, Tafer H, Tu ZJ, Van Tassell CP, Vilella AJ, Williams KP, Yorke JA, Zhang L, Zhang HB, Zhang X, Zhang Y, Reed KM (2010). Multi-platform next-generation sequencing of the domestic turkey (Meleagris gallopavo): genome assembly and analysis. PLOS Biology.

[ref-16] Dames SA, Martinez-Yamout M, De Guzman RN, Dyson HJ, Wright PE (2002). Structural basis for Hif-1 alpha /CBP recognition in the cellular hypoxic response. Proceedings of the National Academy of Sciences of the United States of America.

[ref-17] De Bie T, Cristianini N, Demuth JP, Hahn MW (2006). CAFE: a computational tool for the study of gene family evolution. Bioinformatics.

[ref-18] Edgar RC (2004). MUSCLE: multiple sequence alignment with high accuracy and high throughput. Nucleic Acids Research.

[ref-19] Ekblom R, Wolf JB (2014). A field guide to whole-genome sequencing, assembly and annotation. Evolutionary Applications.

[ref-20] Faraci FM (1991). Adaptations to hypoxia in birds: how to fly high. Annual Review of Physiology.

[ref-21] Finn RD, Bateman A, Clements J, Coggill P, Eberhardt RY, Eddy SR, Heger A, Hetherington K, Holm L, Mistry J, Sonnhammer ELL, Tate J, Punta M (2014). Pfam: the protein families database. Nucleic Acids Research.

[ref-22] Gao G, Zhao X, Li Q, He C, Zhao W, Liu S, Ding J, Ye W, Wang J, Chen Y, Wang H, Li J, Luo Y, Su J, Huang Y, Liu Z, Dai R, Shi Y, Meng H, Wang Q (2016). Genome and metagenome analyses reveal adaptive evolution of the host and interaction with the gut microbiota in the goose. Scientific Reports.

[ref-23] Ge RL, Cai Q, Shen YY, San A, Ma L, Zhang Y, Yi X, Chen Y, Yang L, Huang Y, He R, Hui Y, Hao M, Li Y, Wang B, Ou X, Xu J, Zhang Y, Wu K, Geng C, Zhou W, Zhou T, Irwin DM, Yang Y, Ying L, Bao H, Kim J, Larkin DM, Ma J, Lewin HA, Xing J, Platt RN, Ray DA, Auvil L, Capitanu B, Zhang X, Zhang G, Murphy RW, Wang J, Zhang YP, Wang J (2013). Draft genome sequence of the Tibetan antelope. Nature Communications.

[ref-24] Gregory TR (2019). Animal genome size database. http://www.genomesize.com.

[ref-25] Guigo R, Knudsen S, Drake N, Smith T (1992). Prediction of gene structure. Journal of Molecular Biology.

[ref-26] Haas BJ, Salzberg SL, Zhu W, Pertea M, Allen JE, Orvis J, White O, Buell CR, Wortman JR (2008). Automated eukaryotic gene structure annotation using EVidenceModeler and the program to assemble spliced alignments. Genome Biology.

[ref-27] Hawkes LA, Balachandran S, Batbayar N, Butler PJ, Frappell PB, Milsom WK, Tseveenmyadag N, Newman SH, Scott GR, Sathiyaselvam P, Takekawa JY, Wikelski M, Bishop CM (2011). The trans-Himalayan flights of bar-headed geese (Anser indicus). Proceedings of the National Academy of Sciences of the United States of America.

[ref-28] Huang Y, Li Y, Burt DW, Chen H, Zhang Y, Qian W, Kim H, Gan S, Zhao Y, Li J, Yi K, Feng H, Zhu P, Li B, Liu Q, Fairley S, Magor KE, Du Z, Hu X, Goodman L, Tafer H, Vignal A, Lee T, Kim KW, Sheng Z, An Y, Searle S, Herrero J, Groenen MAM, Crooijmans RPMA, Faraut T, Cai Q, Webster RG, Aldridge JR, Warren WC, Bartschat S, Kehr S, Marz M, Stadler PF, Smith J, Kraus RHS, Zhao Y, Ren L, Fei J, Morisson M, Kaiser P, Griffin DK, Rao M, Pitel F, Wang J, Li N (2013). The duck genome and transcriptome provide insight into an avian influenza virus reservoir species. Nature Genetics.

[ref-29] International Chicken Genome Sequencing Consortium (2004). Sequence and comparative analysis of the chicken genome provide unique perspectives on vertebrate evolution. Nature.

[ref-30] Jarvis ED, Mirarab S, Aberer AJ, Li B, Houde P, Li C, Ho SY, Faircloth BC, Nabholz B, Howard JT, Suh A, Weber CC, Da Fonseca RR, Li J, Zhang F, Li H, Zhou L, Narula N, Liu L, Ganapathy G, Boussau B, Bayzid MS, Zavidovych V, Subramanian S, Gabaldon T, Capella-Gutiérrez S, Huerta-Cepas J, Rekepalli B, Munch K, Schierup M, Lindow B, Warren WC, Ray D, Green RE, Bruford MW, Zhan X, Dixon A, Li S, Li N, Huang Y, Derryberry EP, Bertelsen MF, Sheldon FH, Brumfield RT, Mello CV, Lovell PV, Wirthlin M, Schneider MP, Prosdocimi F, Samaniego JA, Vargas Velazquez AM, Alfaro-Núñez A, Campos PF, Petersen B, Sicheritz-Ponten T, Pas A, Bailey T, Scofield P, Bunce M, Lambert DM, Zhou Q, Perelman P, Driskell AC, Shapiro B, Xiong Z, Zeng Y, Liu S, Li Z, Liu B, Wu K, Xiao J, Yinqi X, Zheng Q, Zhang Y, Yang H, Wang J, Smeds L, Rheindt FE, Braun M, Fjeldsa J, Orlando L, Barker FK, Jønsson KA, Johnson W, Koepfli KP, O’Brien S, Haussler D, Ryder OA, Rahbek C, Willerslev E, Graves GR, Glenn TC, McCormack J, Burt D, Ellegren H, Alström P, Edwards SV, Stamatakis A, Mindell DP, Cracraft J, Braun EL, Warnow T, Jun W, Gilbert MT, Zhang G (2014). Whole-genome analyses resolve early branches in the tree of life of modern birds. Science.

[ref-31] Javed S, Takekawa JY, Douglas DC, Rahmani AR, Kanai Y, Nagendran M, Choudhury BC, Sharma S (2000). Tracking the spring migration of a bar-headed goose (Anser indicus) across the Himalaya with satellite telemetry. Global Environmental Research.

[ref-32] Kalvari I, Nawrocki EP, Argasinska J, Quinones-Olvera N, Finn RD, Bateman A, Petrov AI (2018). Non-coding RNA analysis using the Rfam database. Current Protocols in Bioinformatics.

[ref-33] Kapusta A, Suh A (2017). Evolution of bird genomes-a transposon’s-eye view. Annals of the New York Academy of Sciences.

[ref-34] Kim D, Pertea G, Trapnell C, Pimentel H, Kelley R, Salzberg SL (2013). TopHat2: accurate alignment of transcriptomes in the presence of insertions, deletions and gene fusions. Genome Biology.

[ref-35] Korf I (2004). Gene finding in novel genomes. BMC Bioinformatics.

[ref-36] Kraus RHS, Wink M (2015). Avian genomics: fledging into the wild!. Journal of Ornithology.

[ref-37] Kumar S, Vaidya M (2016). Hypoxia inhibits mesenchymal stem cell proliferation through HIF1 *α*-dependent regulation of P27. Molecular and Cellular Biochemistry.

[ref-38] Lamichhaney S, Fan G, Widemo F, Gunnarsson U, Thalmann DS, Hoeppner MP, Kerje S, Gustafson U, Shi C, Zhang H, Chen W, Liang X, Huang L, Wang J, Liang E, Wu Q, Lee SM, Xu X, Höglund J, Liu X, Andersson L (2016). Structural genomic changes underlie alternative reproductive strategies in the ruff (Philomachus pugnax). Nature Genetics.

[ref-39] Li H, Coghlan A, Ruan J, Coin LJ, Heriche JK, Osmotherly L, Li RQ, Liu T, Zhang Z, Bolund L, Wong G, Zheng W, Dehal P, Wang J, Durbin R (2006). TreeFam: a curated database of phylogenetic trees of animal gene families. Nucleic Acids Research.

[ref-40] Li S, Li B, Cheng C, Xiong Z, Liu Q, Lai J, Carey HV, Zhang Q, Zheng H, Wei S, Zhang H, Chang L, Liu S, Zhang S, Yu B, Zeng X, Hou Y, Nie W, Guo Y, Chen T, Han J, Wang J, Wang J, Chen C, Liu J, Stambrook PJ, Xu M, Zhang G, Gilbert MT, Yang H, Jarvis ED, Yu J, Yan J (2014). Genomic signatures of near-extinction and rebirth of the crested ibis and other endangered bird species. Genome Biology.

[ref-41] Liu D, Zhang G, Li F, Ma T, Lu J, Qian F (2017). A revised species population estimate for the bar-headed goose (Anser indicus). Avian Research.

[ref-42] Lu L, Chen Y, Wang Z, Li X, Chen W, Tao Z, Shen J, Tian Y, Wang D, Li G, Chen L, Chen F, Fang D, Yu L, Sun Y, Ma Y, Li J, Wang J (2015). The goose genome sequence leads to insights into the evolution of waterfowl and susceptibility to fatty liver. Genome Biology.

[ref-43] Majoros WH, Pertea M, Salzberg SL (2004). TigrScan and GlimmerHMM: two open source ab initio eukaryotic gene-finders. Bioinformatics.

[ref-44] McCracken KG, Barger CP, Sorenson MD (2010). Phylogenetic and structural analysis of the HbA (alphaA/betaA) and HbD (alphaD/betaA) hemoglobin genes in two high-altitude waterfowl from the Himalayas and the Andes: bar-headed goose (Anser indicus) and Andean goose (Chloephaga melanoptera). Molecular Phylogenetics and Evolution.

[ref-45] Monge C, Leon-Velarde F (1991). Physiological adaptation to high altitude: oxygen transport in mammals and birds. Physiological Reviews.

[ref-46] Mulder N, Apweiler R (2007). Inter pro and inter pro scan: tools for protein sequence classification and comparison. Methods in Molecular Biology.

[ref-47] Nakao K, Itoh H, Saito Y, Mukoyama M, Ogawa Y (1996). The natriuretic peptide family. Current Opinion in Nephrology and Hypertension.

[ref-48] Natarajan C, Jendroszek A, Kumar A, Weber RE, Tame JRH, Fago A, Storz JF (2018). Molecular basis of hemoglobin adaptation in the high-flying bar-headed goose. PLOS Genetics.

[ref-49] Nawrocki EP, Kolbe DL, Eddy SR (2009). Infernal 1.0: inference of RNA alignments. Bioinformatics.

[ref-50] Ogata H, Goto S, Sato K, Fujibuchi W, Bono H, Kanehisa M (1999). KEGG: kyoto encyclopedia of genes and genomes. Nucleic Acids Research.

[ref-51] O’Leary NA, Wright MW, Brister JR, Ciufo S, Haddad D, McVeigh R, Rajput B, Robbertse B, Smith-White B, Ako-Adjei D, Astashyn A, Badretdin A, Bao Y, Blinkova O, Brover V, Chetvernin V, Choi J, Cox E, Ermolaeva O, Farrell CM, Goldfarb T, Gupta T, Haft D, Hatcher E, Hlavina W, Joardar VS, Kodali VK, Li W, Maglott D, Masterson P, McGarvey KM, Murphy MR, O’Neill K, Pujar S, Rangwala SH, Rausch D, Riddick LD, Schoch C, Shkeda A, Storz SS, Sun H, Thibaud-Nissen F, Tolstoy I, Tully RE, Vatsan AR, Wallin C, Webb D, Wu W, Landrum MJ, Kimchi A, Tatusova T, DiCuccio M, Kitts P, Murphy TD, Pruitt KD (2016). Reference sequence (RefSeq) database at NCBI: current status, taxonomic expansion, and functional annotation. Nucleic Acids Research.

[ref-52] Ottenburghs J, Megens HJ, Kraus RHS, Madsen O, Van Hooft P, Van Wieren SE, Crooijmans RPMA, Ydenberg RC, Groenen MAM, Prins HHT (2016). A tree of geese: a phylogenomic perspective on the evolutionary history of True Geese. Molecular Phylogenetics and Evolution.

[ref-53] Ottenburghs J, Megens HJ, Kraus RHS, Van Hooft P, Van Wieren SE, Crooijmans RPMA, Ydenberg RC, Groenen MAM, Prins HHT (2017). A history of hybrids? Genomic patterns of introgression in the True Geese. BMC Evolutionary Biology.

[ref-54] Paajanen P, Kettleborough G, Lopez-Girona E, Giolai M, Heavens D, Baker D, Lister A, Wilde G, Hein I, Macaulay I, Bryan GJ, Clark MD (2019). A critical comparison of technologies for a plant genome sequencing project. Gigascience.

[ref-55] Parra G, Bradnam K, Korf I (2007). CEGMA: a pipeline to accurately annotate core genes in eukaryotic genomes. Bioinformatics.

[ref-56] Potter SC, Luciani A, Eddy SR, Park Y, Lopez R, Finn RD (2018). HMMER web server: 2018 update. Nucleic Acids Research.

[ref-57] Projecto-Garcia J, Chandrasekhar N, Hideaki M, Roy EW, Angela F, Zachary AC, Robert D, Jimmy AM, Christopher CW, Jay FS (2013). Repeated elevational transitions in hemoglobin function during the evolution of Andean hummingbirds. Proceedings of the National Academy of Sciences of the United States of America.

[ref-58] Pujolar JM, Dalen L, Olsen RA, Hansen MM, Madsen J (2018). First de novo whole genome sequencing and assembly of the pink-footed goose. Genomics.

[ref-59] Qiu Q, Zhang G, Ma T, Qian W, Wang J, Ye Z, Cao C, Hu Q, Kim J, Larkin DM, Auvil L, Capitanu B, Ma J, Lewin HA, Qian X, Lang Y, Zhou R, Wang L, Wang K, Xia J, Liao S, Pan S, Lu X, Hou H, Wang Y, Zang X, Yin Y, Ma H, Zhang J, Wang Z, Zhang Y, Zhang D, Yonezawa T, Hasegawa M, Zhong Y, Liu W, Zhang Y, Huang Z, Zhang S, Long R, Yang H, Wang J, Lenstra JA, Cooper DN, Wu Y, Wang J, Shi P, Wang J, Liu J (2012). The yak genome and adaptation to life at high altitude. Nature Genetics.

[ref-60] Qu Y, Tian S, Han N, Zhao H, Gao B, Fu J, Cheng Y, Song G, Ericson PG, Zhang YE, Wang D, Quan Q, Jiang Z, Li R, Lei F (2015). Genetic responses to seasonal variation in altitudinal stress: whole-genome resequencing of great tit in eastern Himalayas. Scientific Reports.

[ref-61] Qu Y, Zhao H, Han N, Zhou G, Song G, Gao B, Tian S, Zhang J, Zhang R, Meng X, Zhang Y, Zhang Y, Zhu X, Wang W, Lambert D, Ericson PG, Subramanian S, Yeung C, Zhu H, Jiang Z, Li R, Lei F (2013). Ground tit genome reveals avian adaptation to living at high altitudes in the Tibetan plateau. Nature Communications.

[ref-62] Schattner P, Brooks AN, Lowe TM (2005). The tRNAscan-SE, snoscan and snoGPS web servers for the detection of tRNAs and snoRNAs. Nucleic Acids Research.

[ref-63] Scott GR (2011). Elevated performance: the unique physiology of birds that fly at high altitudes. Journal of Experimental Biology.

[ref-64] Scott GR, Egginton S, Richards JG, Milsom WK (2009). Evolution of muscle phenotype for extreme high altitude flight in the bar-headed goose. Proceedings of the Royal Society B: Biological Sciences.

[ref-65] Scott GR, Hawkes LA, Frappell PB, Butler PJ, Bishop CM, Milsom WK (2015). How bar-headed geese fly over the Himalayas. Physiology.

[ref-66] Scott GR, Milsom WK (2007). Control of breathing and adaptation to high altitude in the bar-headed goose. American Journal of Physiology, Regulatory Integrative and Comparative Physiology.

[ref-67] Shapiro MD, Kronenberg Z, Li C, Domyan ET, Pan H, Campbell M, Tan H, Huff CD, Hu H, Vickrey AI, Nielsen SCA, Stringham SA, Hu H, Willerslev E, Gilbert MTP, Yandell M, Zhang G, Wang J (2013). Genomic diversity and evolution of the head crest in the rock pigeon. Science.

[ref-68] Simao FA, Waterhouse RM, Ioannidis P, Kriventseva EV, Zdobnov EM (2015). BUSCO: assessing genome assembly and annotation completeness with single-copy orthologs. Bioinformatics.

[ref-69] Smit A, Hubley R (2018). RepeatModeler-1.0.11. https://repeatmasker.org/RepeatModeler/.

[ref-70] Snyder GK, Byers RL, Kayar SR (1984). Effects of hypoxia on tissue capillarity in geese. Respiratory Physiology.

[ref-71] Stanke M, Morgenstern B (2005). AUGUSTUS: a web server for gene prediction in eukaryotes that allows user-defined constraints. Nucleic Acids Research.

[ref-72] Sun W, Quan N, Wang L, Yang H, Chu D, Liu Q, Zhao X, Leng J, Li J (2016). Cardiac-specific deletion of the Pdha1 gene sensitizes heart to toxicological actions of ischemic stress. Toxicological Sciences.

[ref-73] Takekawa JY, Heath SR, Douglas DC, Perry WM, Javed S, Newman SH, Suwal RN, Rahmani AR, Choudhury BC, Prosser DJ, Yan B, Hou Y, Batbayar N, Natsagdorj T, Bishop C, Butler P, Frappell P, Milsom W, Scott G, Hawkes L, Wikelski M (2009). Geographic variation in bar-headed geese Anser indicus: connectivity of wintering areas and breeding grounds across a broad front. Wildfowl.

[ref-74] Tarailo-Graovac M, Chen N (2009). Using RepeatMasker to identify repetitive elements in genomic sequences. Current Protocols in Bioinformatics.

[ref-75] Trapnell C, Williams BA, Pertea G, Mortazavi A, Kwan G, Van Baren MJ, Salzberg SL, Wold BJ, Pachter L (2010). Transcript assembly and quantification by RNA-Seq reveals unannotated transcripts and isoform switching during cell differentiation. Nature Biotechnology.

[ref-76] Tucker VA (1968). Respiratory physiology of house sparrows in relation to high-altitude flight. Journal of Experimental Biology.

[ref-77] Ward S, Bishop CM, Woakes AJ, Butler PJ (2002). Heart rate and the rate of oxygen consumption of flying and walking barnacle geese (Branta leucopsis) and bar-headed geese (Anser indicus). Journal of Experimental Biology.

[ref-78] Warren WC, Clayton DF, Ellegren H, Arnold AP, Hillier LW, Kunstner A, Searle S, White S, Vilella AJ, Fairley S, Heger A, Kong L, Ponting CP, Jarvis ED, Mello CV, Minx P, Lovell P, Velho TA, Ferris M, Balakrishnan CN, Sinha S, Blatti C, London SE, Li Y, Lin YC, George J, Sweedler J, Southey B, Gunaratne P, Watson M, Nam K, Backström N, Smeds L, Nabholz B, Itoh Y, Whitney O, Pfenning AR, Howard J, Völker M, Skinner BM, Griffin DK, Ye L, McLaren WM, Flicek P, Quesada V, Velasco G, Lopez-Otin C, Puente XS, Olender T, Lancet D, Smit AF, Hubley R, Konkel MK, Walker JA, Batzer MA, Gu W, Pollock DD, Chen L, Cheng Z, Eichler EE, Stapley J, Slate J, Ekblom R, Birkhead T, Burke T, Burt D, Scharff C, Adam I, Richard H, Sultan M, Soldatov A, Lehrach H, Edwards SV, Yang SP, Li X, Graves T, Fulton L, Nelson J, Chinwalla A, Hou S, Mardis ER, Wilson RK (2010). The genome of a songbird. Nature.

[ref-79] Weisenfeld NI, Kumar V, Shah P, Church DM, Jaffe DB (2017). Direct determination of diploid genome sequences. Genome Research.

[ref-80] Yang Z (2007). PAML 4: phylogenetic analysis by maximum likelihood. Molecular Biology and Evolution.

[ref-81] Zhan X, Pan S, Wang J, Dixon A, He J, Muller MG, Ni P, Hu L, Liu Y, Hou H, Chen Y, Xia J, Luo Q, Xu P, Chen Y, Liao S, Cao C, Gao S, Wang Z, Yue Z, Li G, Yin Y, Fox NC, Wang J, Bruford MW (2013). Peregrine and saker falcon genome sequences provide insights into evolution of a predatory lifestyle. Nature Genetics.

[ref-82] Zhang G, Li C, Li Q, Li B, Larkin DM, Lee C, Storz JF, Antunes A, Greenwold MJ, Meredith RW, Ödeen A, Cui J, Zhou Q, Xu L, Pan H, Wang Z, Jin L, Zhang P, Hu H, Yang W, Hu J, Xiao J, Yang Z, Liu Y, Xie Q, Yu H, Lian J, Wen P, Zhang F, Li H, Zeng Y, Xiong Z, Liu S, Zhou L, Huang Z, An N, Wang J, Zheng Q, Xiong Y, Wang G, Wang B, Wang J, Fan Y, Da Fonseca RR, Alfaro-Núñez A, Schubert M, Orlando L, Mourier T, Howard JT, Ganapathy G, Pfenning A, Whitney O, Rivas MV, Hara E, Smith J, Farré M, Narayan J, Slavov G, Romanov MN, Borges R, Machado JP, Khan I, Springer MS, Gatesy J, Hoffmann FG, Opazo JC, Håstad O, Sawyer RH, Kim H, Kim KW, Kim HJ, Cho S, Li N, Huang Y, Bruford MW, Zhan X, Dixon A, Bertelsen MF, Derryberry E, Warren W, Wilson RK, Li S, Ray DA, Green RE, O’Brien SJ, Griffin D, Johnson WE, Haussler D, Ryder OA, Willerslev E, Graves GR, Alström P, Fjeldså J, Mindell DP, Edwards SV, Braun EL, Rahbek C, Burt DW, Houde P, Zhang Y, Yang H, Wang J, Jarvis ED, Gilbert MT, Wang J, Avian Genome Consortium (2014). Comparative genomics reveals insights into avian genome evolution and adaptation. Science.

[ref-83] Zhang G, Rahbek C, Graves GR, Lei F, Jarvis ED, Gilbert MT (2015). Genomics: bird sequencing project takes off. Nature.

[ref-84] Zhang Q, Han X, Kraus RHS, Yang L, Fan L, Ye Y, Tao Y (2016a). The expression plasticity of hypoxia related genes in high altitude and plains Nanorana parkeri populations. Asian Herpetological Research.

[ref-85] Zhang Q, Han X, Ye Y, Kraus RHS, Fan L, Yang L, Tao Y (2016b). Expression of HIF-1 alpha and its target genes in the Nanorana parkeri heart: implications for high altitude adaptation. Asian Herpetological Research.

[ref-86] Zhou C, James JG, Xu Y, Tu H, He X, Wen Q, Price M, Yang N, Wu Y, Ran J, Meng Y, Yue B (2019). Genome-wide analysis sheds light on the high-altitude adaptation of the buff-throated partridge (Tetraophasis szechenyii). Molecular Genetics and Genomics.

[ref-87] Zhu X, Guan Y, Signore AV, Natarajan C, DuBay SG, Cheng Y, Han N, Song G, Qu Y, Moriyama H, Hoffmann FG, Fago A, Lei F, Storz JF (2018). Divergent and parallel routes of biochemical adaptation in high-altitude passerine birds from the Qinghai-Tibet Plateau. Proceedings of the National Academy of Sciences of the United States of America.

