# Supplementary figures and images for: First de novo whole genome sequencing and assembly of the bar-headed goose
Source: PeerJ. 2020 Apr 6;8:e8914. doi: 10.7717/peerj.8914 (PMC7144584; doi:10.7717/peerj.8914)

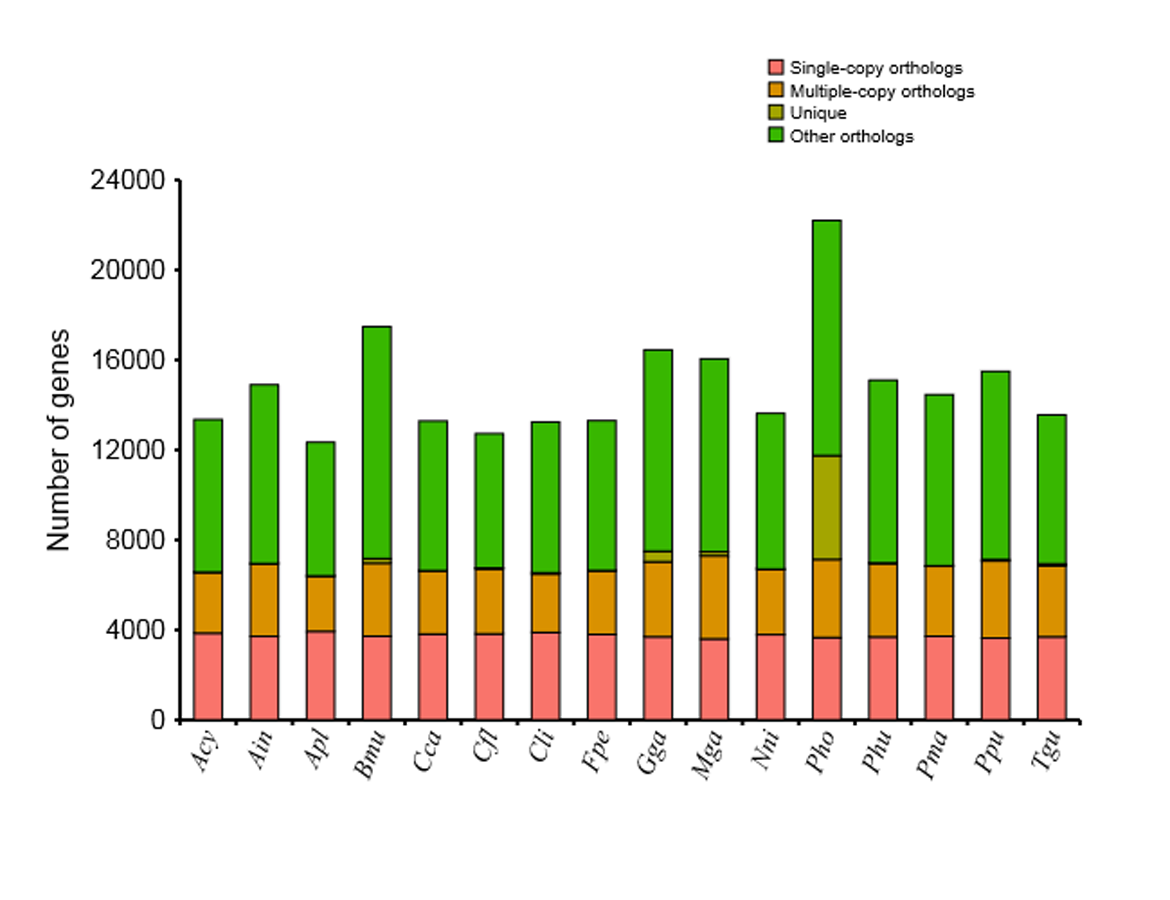

Supplement: Figure S1 — Ain: bar-headed goose. Acy: swan goose. Nni: crested ibis. Apl: mallard. Gga: red junglefowl. Mga: turkey. Cca: common cuckoo. Cli: rock pigeon. Phu: ground tit. Cfl: bananaquit. Pma: great tit. Tgu: zebra finch. Ppu: ruff. Fpe: peregrine falcon. Bmu: yak. Pho: tibetan antelope. [file peerj-08-8914-s001.png]
