# Supplementary material for: First de novo whole genome sequencing and assembly of the bar-headed goose
Source: PeerJ. 2020 Apr 6;8:e8914. doi: 10.7717/peerj.8914 (PMC7144584; doi:10.7717/peerj.8914)
Supplement: Table S1 [file peerj-08-8914-s002.docx]

Table S1 **Summary statistics for the sequencing data.**

|  | Insert size (bp) | Total data (G) | Read length (bp) | Sequence coverage (X) |
| --- | --- | --- | --- | --- |
| Total | 600 | 124 | 150 | 103 |
