# Supplementary material for: First de novo whole genome sequencing and assembly of the bar-headed goose
Source: PeerJ. 2020 Apr 6;8:e8914. doi: 10.7717/peerj.8914 (PMC7144584; doi:10.7717/peerj.8914)
Supplement: Table S2 [file peerj-08-8914-s003.docx]

Table S2 **GC content of the Bar-headed goose genome.**

|  | Number (bp) | % of genome |
| --- | --- | --- |
| A | 323,672,102 | 28.32% |
| T | 324,164,544 | 28.36% |
| C | 233,205,802 | 20.40% |
| G | 233,453,062 | 20.42% |
| N | 28,602,010 | 2.50% |
| GC | 466,658,864 | 41.87% |
| Total (bp) | 1,143,097,520 | - |
