# Supplementary material for: First de novo whole genome sequencing and assembly of the bar-headed goose
Source: PeerJ. 2020 Apr 6;8:e8914. doi: 10.7717/peerj.8914 (PMC7144584; doi:10.7717/peerj.8914)
Supplement: Table S3 — Complete: more than 70% of the core genes were assembled. Complete + partial: partial of the core genes were assembled. #Prots: the number of the assembled core genes. %Completeness: the ratio of the assembled core genes / the whole core gene sets. [file peerj-08-8914-s004.docx]

Table S3 **The CEGMA assessment results of the completeness of genome assembly.**

|  | Complete | | Complete + partial | |
| --- | --- | --- | --- | --- |
| species | #Prots | %Completeness | #Prots | %Completeness |
| Bar-headed goose | 188 | 75.81 | 211 | 85.08 |

Complete: more than 70% of the core genes were assembled. Complete + partial: part of the core genes were assembled. #Prots: the number of the assembled core genes. %Completeness: the ratio of the assembled core genes / the whole core gene sets.
