# Supplementary material for: First de novo whole genome sequencing and assembly of the bar-headed goose
Source: PeerJ. 2020 Apr 6;8:e8914. doi: 10.7717/peerj.8914 (PMC7144584; doi:10.7717/peerj.8914)
Supplement: Table S4 [file peerj-08-8914-s005.docx]

Table S4 **Annotation of repeated sequences.**

| Tools | Repeat Size (bp) | % of genome |
| --- | --- | --- |
| TRF | 11,325,101 | 0.99 |
| RepeatMasker | 89,218,647 | 7.80 |
| RepeatProteinMask | 49,022,443 | 4.29 |
| Total | 101,855,129 | 8.91 |

TRF: Tandem Repeats Finder.
